# Characterization of Organic Layer in Oil Carbonate Reservoir Rocks and its Effect on Microscale Wetting Properties

**DOI:** 10.1038/s41598-019-47139-y

**Published:** 2019-07-23

**Authors:** Anastasia Ivanova, Nikolai Mitiurev, Alexey Cheremisin, Anton Orekhov, Roman Kamyshinsky, Alexander Vasiliev

**Affiliations:** 1 Skolkovo Institute of Science and Technology, Moscow, 121205 Russia; 2 Curtin University, Perth, WA 6845 Australia; 3 Emanuel Institute of Biochemical Physics, Russian Academy of Sciences, Moscow, 119334 Russia; 4 National Research Centre “Kurchatov Institute”, Moscow, 123182 Russia

**Keywords:** Geochemistry, Geochemistry

## Abstract

Effective production of oil from carbonate reservoirs often requires the application of improved oil recovery technologies such as waterflooding. However, conventional waterflooding in carbonates usually results in low hydrocarbon recovery as most of these formations exhibit a complex pore throats structure and are mostly oil-wet. Therefore, improved insight into the causes of hydrophobic wetting behavior of such reservoirs is important for understanding the fluid distribution, displacement and enhancing recovery processes. The characterization of fluid-rock interactions is, however, challenging with existing laboratory methods, which are typically based on macroscale (mm) observations. In this experimental study, an advanced imaging technique, namely environmental scanning electron microscope, was applied for the comprehensive investigation of microscale (µm) wettability variations in carbonate rocks covered with organic layers. For the first time, the presence of organic layers on the sample was proved using energy dispersive X-ray mapping. Furthermore, the chemical bond of this layer and carbonate rock surfaces was determined using the transmission electron microscopy and electron energy-loss spectroscopy. The thickness of layer was estimated by using image processing software. These findings show that the application of combined microscopic techniques reveals important details about the reason of hydrophobic wetting properties of real carbonate rocks.

## Introduction

Carbonate reservoirs, particularly limestones and dolomites, represent more than half of the world’s oil resources^1^. However, primary oil recovery from such reservoirs is often poor, and require the use of improved oil recovery (IOR) techniques, such as water or gas flooding^2–4^. Efficient oil recovery from carbonate reservoirs by waterflooding strongly depends on initial wetting properties of rocks, especially for fractured reservoirs where the main mechanism of oil displacement through the fractured networks is capillary force effect^5^. It is well documented in the literature that the majority of carbonate reservoirs have mixed or oil-wet wetting properties due to the presence of organic components adsorbed physically or chemically from crude oil on rock surfaces^6–10^. Indeed, Buckley *et al*.^11^ demonstrated that asphaltene and resin fractions from crude oil have a tendency to adsorb on solid rock surfaces, causing wettability alteration of rock samples.

Furthermore, Madsen *et al*.^12^ observed strong adsorption of carboxylic acids (benzoic acid) from a hydrocarbon phase on synthetic calcite minerals. The authors indicated that the adsorption value on calcite (*Г*max = 7.35 ± 0.15 (*µ*mol/m^2^)) is higher than on other minerals, such as kaolinite (3.14 ± 0.25 (*µ*mol/m^2^)) or quartz (2.76 ± 0.19 (*µ*mol/m^2^)). Strong adsorption capacity of saturated fatty acids (e.g. palmitic, oleic and octanoic acids) on carbonate minerals was also reported by Thomas *et al*.^13^. The authors pointed out that the mechanism of stable bond between carbonate surface and acids is based on the attached carboxylate groups of adsorbates, which react with calcium ions and form close-packed hydrocarbon layers above the mineral surface. The authors also mentioned that these layers could modify the wettability of carbonate minerals towards more oil-wet, and thus, could lead to oil recovery reduction.

In terms of oil recovery, wettability is a key parameter to control injected and produced fluids arrangement in the porous media, which in turn influences the properties of multiphase flow of water, gas and oil^14–16^. Therefore, the study of wettability properties plays a major role in reservoir characterization and development of either sandstones or carbonates. Traditionally, wettability studies are conducted with core-scale measurements using USBM (U.S. Bureau of Mines)^17,18^, Amott^19^, and contact angle methods^20^. The popularity of these methods stems from their simplicity and cost-effectiveness. However, direct investigation of the rock-fluid interactions by these methods is limited, as they yield the average wettability indexes on macroscale (mm), and cannot account for rock surface roughness, pore structure or chemical rock composition, which strongly affect fluids flow properties^21^. The average wettability has been measured for many sandstone and carbonate rocks^22–27^, whereas there is a serious lack of information about microscale wettability characterization of pores, where the majority of hydrocarbons resides.

We thus aim to obtain a deeper insight into micro-scale wetting properties inside pores with the use of the advanced microscopic technique – environmental scanning electron microscopy (ESEM). The key element of our work is the investigation of the nature of the layers adsorbed onto real carbonate surfaces and their influence on wetting properties. This is achieved using cryo-focused ion beam (Cryo-FIB) sample preparation approach followed by the implementation of scanning/transmission electron microscopy (S/TEM) and electron energy loss spectroscopy (EELS). The studied core material was taken from the oil zone of a low-permeability carbonate reservoir. The combination of ESEM and Cryo-FIB methods together with S/TEM reveals important details about the micro and nano-pore structure of rocks, which further allow both laboratory and field scale models of oil recovery and potential of carbon dioxide storage to be developed^28,29^. Indeed, Deglint *et al*.^30^ carried out the micro-wettability (µm) measurements using ESEM with the middle Bakken samples and inferred that these results could be used for more accurate estimation of multi-phase flow parameters (e.g. relative permeability, capillary pressure), which in turn would improve primary and secondary oil recovery processes. It is interesting to point out that the fluid distributions through rock samples at reservoir temperature and pressure can be also obtained using the non-invasive microtomography (µCT) method^31^. For instance, Andrew *et al*.^31^ measured the pore-scale contact angles of super-critical (sc) CO_2_ – brine – calcite systems based on µCT sample images, showing that obtained results could be used as an input for building the pore-scale models.

In this study, the experimental setup with ESEM and Cryo-FIB techniques was established in order to avoid the damaging of organic layers on the surface of carbonate rocks during sample preparation. Cryo-FIB approach was implemented to identify the organic layer and assess its influence on micro - wetting properties of carbonate rocks. In the first step, the energy dispersive X-ray spectroscopy (EDXS) technique was used to analyze the elemental composition of the surface and organic layer. The wettability of initial and toluene-extracted samples of carbonate rocks was examined using live-imaging ESEM experiments. Once water droplets were formed on samples surfaces, contact angles were calculated. The microstructure of sample areas containing organic layers was examined using S/TEM and EELS. Therefore, the current work based on the latest advances in ESEM^28,30,32–35^ and Cryo-FIB^36,37^ focuses on identification and characterization of organic layers adsorbed on real carbonate surfaces on micro (µm) - and nanoscales (nm).

## Results

### Micro-wettability investigation of the initial carbonate sample

**Figure 1:**
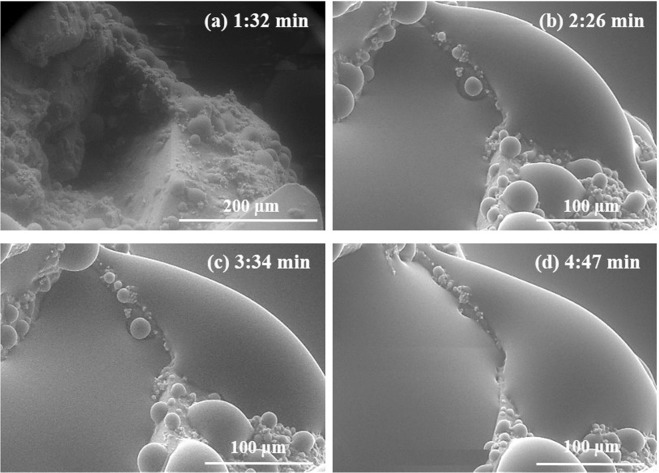
Water droplets formation on the initial carbonate surface in dependence of time from 1:32 minutes (**a**) to 4:47 minutes (**d**). The sample temperature and chamber pressure were kept around 0 °C and 850 Pa respectively for every experiment.

To calculate contact angles, we performed the time-lapse analysis of droplets formation in order to select the moment when droplets have just appeared (as suggested in previous studies that describe ESEM method^30,35^). An example for the initial carbonate sample is shown in Fig. 1, which illustrates that after a while droplets start to merge into each other, forming a large droplet that covers all surface.

As can be observed from Fig. 1a, water droplets appear on the surface at 1:32 minutes and then continue to grow until almost all of them coalesce into one large droplet (Fig. 1d). Such coacervating changes the contact line between phases and impedes contact angle calculation. Thus, for the appropriate contact angles evaluation, we selected water condensation time of around 1:30 minutes.

**Figure 2:**
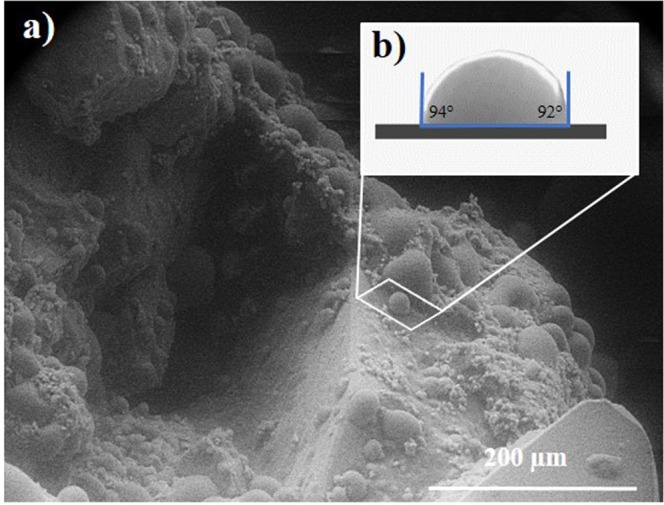
(**a**) ESEM image of initial carbonate rock with formed water droplets. (**b**) Extracted from (**a**) droplet with measured dimensions – height of 0.24 µm and radii of 0.22 µm (left) and 0.23 µm (right). The average contact angle is 93 ± 1°.

Once thermobaric conditions and time for droplets growth have been experimentally found, the droplets were cut from the imaged region (Fig. 1a) and analyzed using image processing software ImageJ^38^ (see contact angle evaluation procedure) to calculate contact angle values (Fig. 2b).

The measured micro - droplet height was 0.24 µm and radii from right side and left side equaled to 0.23 µm and 0.22 µm respectively. Then, Eqs (1) and (2) gave average contact angle of 93 ± 1°. This value corresponded to hydrophobic wetting behavior of the surface.

### Micro-wettability investigation of the sample after toluene extraction

Using the same procedure further experiments were conducted with the sample after toluene extraction. Visualization and EDXS analysis of such sample allowed to determine organic layer locations on the surface, and its influence on micro-scale wetting properties.

**Figure 3:**
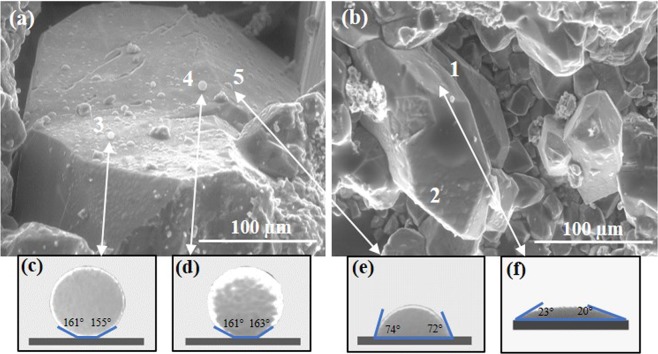
(**a**,**b**) ESEM images with formed water droplets on carbonate surface after solvent extraction; (**c**–**f**) Extracted droplet profiles from picture (**a**,**b**) respectively with calculated contact angles; (**c**–**e**) droplet images represent mixed wetting properties of carbonate surface with two different types of contact angle – 160 ± 3° and 73 ± 1°; (**f**) image refers to hydrophilic wetting properties with the average contact angle of 22 ± 2°. X-ray analyses were performed in points 3, 4, 5 in (**a**) and 1, 2 in (**b**) ESEM images. The sample temperature and chamber pressure were kept around 0 °C and 850 Pa respectively.

The sample was cleaned with kerosene and toluene, allowing crude oil to be removed from the sample and broken to uncover fresh pore surfaces, and then investigated (Fig. 3).

The contact angles between distilled water droplets and carbonate surface were calculated using the measured droplet dimensions. Four water droplets were analyzed – two were very similar with an average contact angle of 160 ± 3° (Fig. 3c,d), one had dimensions of 0.19 µm (height) and 0.51 µm (diameter) with the average contact angle of 73 ± 1° (Fig. 3e), and the last droplet had dimensions of 0.07 µm (height) and 0.74 µm (diameter) with the average contact angle of 22 ± 2° (Fig. 3f). Furthermore, focusing in Fig. 3b, it can be observed that solvent extraction removed all initial oil from sample pores, making them more water-wet with the average contact angle 22 ± 2°, which wasn’t observed for samples before the extractions (Fig. 2). Presence of several distinct contact angles, which represent hydrophobic and hydrophilic zones of surface, suggests that the sample’s micro-wettability is mixed.

**Figure 4:**
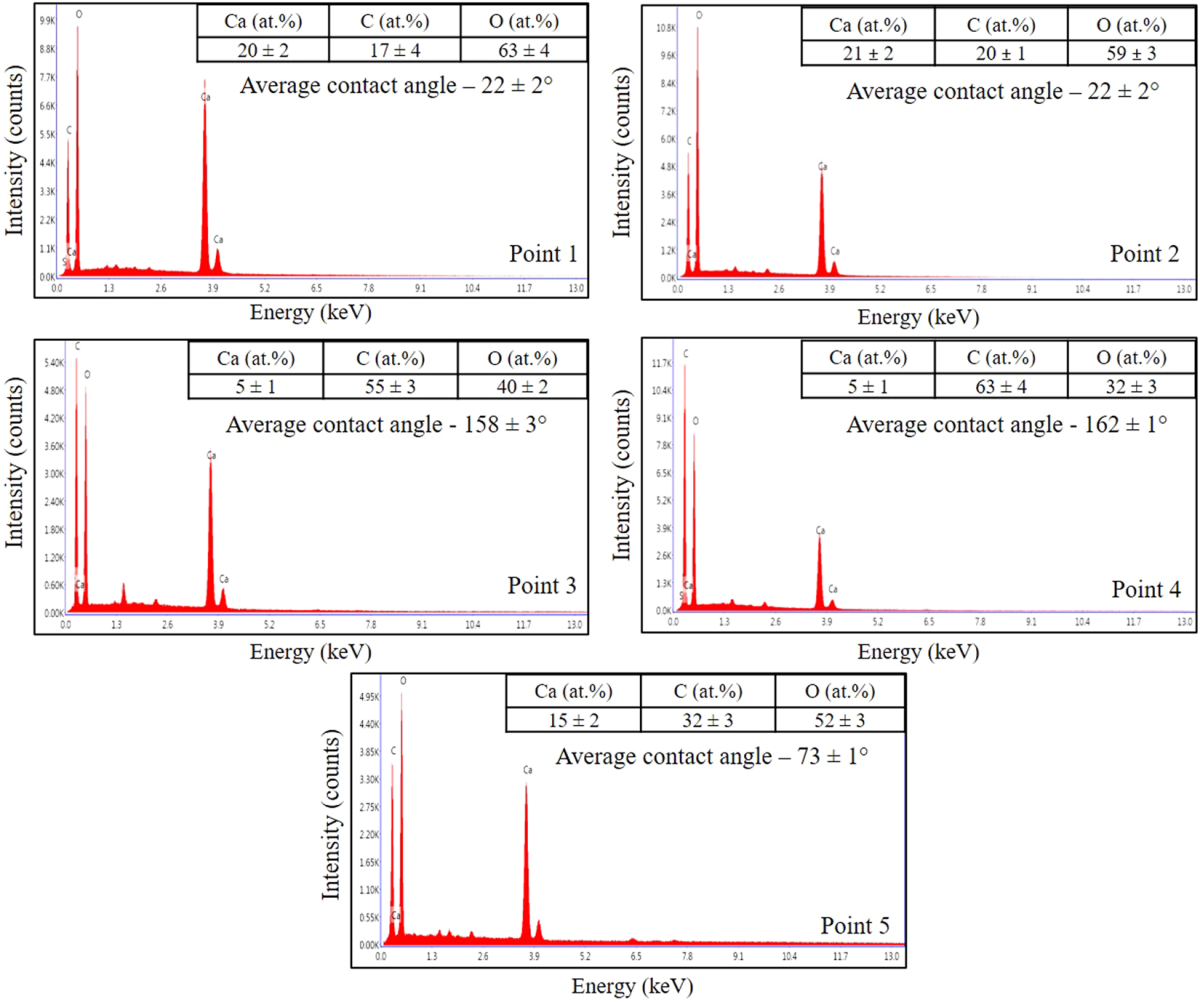
EDX spectra of different points on the surface, showing element concentrations. Points 1 and 2 represent surfaces with average contact angle of 22 ± 2°; points 3 and 4–158 ± 3° and 162 ± 1° respectively; point 5–73 ± 1°. At points 3 and 4 the large amount of carbon − 55 ± 3 at.% and 63 ± 4 at.% was obtained respectively.

Additionally, EDX analysis reveals the type of surface to which water droplets were adhered. Indeed, analysis of the surface which showed the non-oil-wet nature (Fig. 3b points 1 and 2), showed the pure calcite (CaCO_3_) with less than 1% impurities, while elemental estimation of oil-wet surfaces (Fig. 3a points 3 and 4), demonstrated strong carbon signal (Fig. 4).

Calcite (CaCO_3_) was determined at points 1 and 2 (Fig. 4) using a simple proportion between concertation of elements:

$${\rm{Ca}}:{\rm{C}}:{\rm{O}}=20:17:63 \sim 1:1:3$$

However, at points 3 and 4 no molecular formula was determined using this ratio due to the high amount of carbon, which exceeded the necessary amount for the calcite. As such, elemental composition, captured at points 3 and 4, confirmed the presence of the additional carbon, which represented the adsorbed organic layer on calcite.

### Characterization of the organic thin layer

To demonstrate the presence of the organic layer on the carbonate surface, we studied carbonate sample clump after toluene treatment. Analysis of the sample after toluene extraction allowed the remaining organic layer to be determined without the influence of oil. The location of the organic layer on the sample was identified using the results for micro-wettability study and supported by EDXS experiments with the cross-section specimen. The sample surface, which had hydrophobic wetting properties (Fig. 3a), was cut by Cryo-FIB technique (see the experimental section). Then, S/TEM, EDXS, and EELS analyses were carried out to characterize microstructure of the sample, elemental composition, and thickness of the organic layer. This part of the study illustrates the most innovative and complicated procedures in micro- and nanoscales experiments presented in this work.

**Figure 5:**
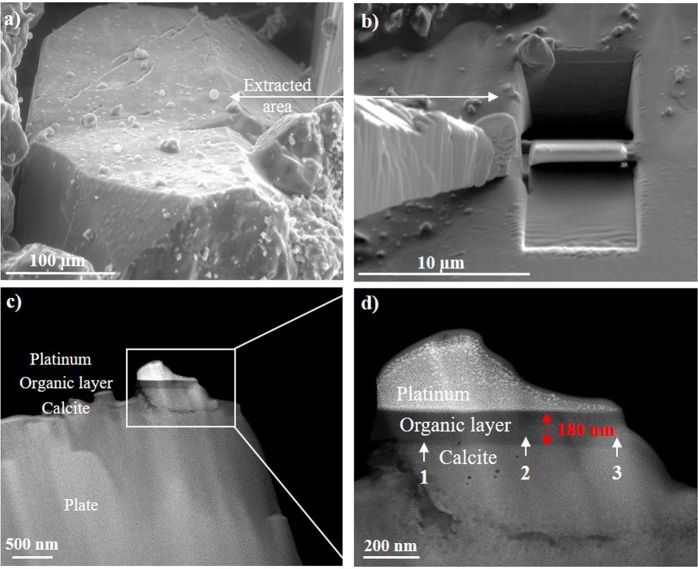
(**a**) ESEM image of the sample surface after toluene cleaning with formed water droplets. (**b**) The sample area from (**a**) after performing FIB milling procedure. (**c**) HAADF-STEM image of extracted cross section of the sample with platinum layer on the top for sample protection. (**d**) Zoom in of the sample in (**c**) illustrating that calcite was covered by organic layer. The measured thickness of the organic layer equaled to 180 ± 12 nm.

Owing to the crisp contrast between phases (Fig. 5d), the thickness of the organic layer was estimated by measuring the length of the dark layer between platinum and calcite. Using this criterion, the estimated thickness of the organic layer was 180 ± 12 nm.

**Figure 6:**
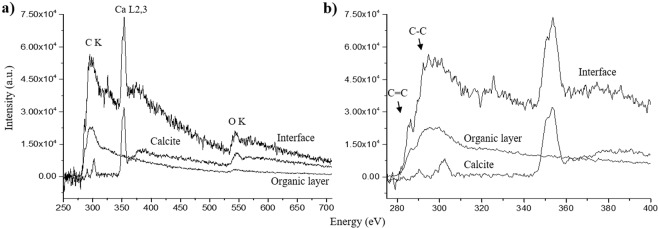
(**a**) The EELS spectra from calcite, organic layer and the interface between them. (**b**) The zoom in of EELS spectrum from picture (**a**) representing the energy interval from 275 eV to 400 eV. The EELS spectrum from calcite illustrates two narrow peaks at 290 eV (C=O) and 299 eV (C-O), and peaks, which correspond to calcium and oxygen at 351 eV and 548 eV respectively. The EELS spectrum from the interface between sample and organic layer shows the C-K edge shoulder at 285 eV and one broad peak at 292 eV, which indicate the existence of amorphous carbon. Peaks at 351 eV and 548 eV confirm the presence of calcium and oxygen.

To determine the chemical bond between the organic layer and calcite, we compared the EELS spectra from calcite, organic layer, and interface between calcite and organic layer (Fig. 6). In the EELS spectrum from calcite, the carbon K edge (C-K) indicates two narrow peaks at 290 eV and 299 eV, which are well-defined as C=O (1s → π*) and C-O (1s → σ*) bonds of carbonate ion respectively^39–41^. The Ca *L*_2,3_ and O-K edges located at 351 eV and 548 eV were also identified.

Focusing on the EELS spectrum from the organic layer, two peaks can be identified – one small sharp peak at 285 eV and a broader peak at 291.5–292 eV, which correspond to amorphous carbon^39^. A small O-K edge located at 547 eV was observed, indicating that oxygen was presented in the organic layer. In comparison with EELS spectra from calcite and from the interface between organic layer and calcite, Ca *L*2,3 edge did not appear.

The EELS spectrum from the interface between organic layer and calcite represents one tiny shoulder at 285 eV and one broad peak at 292 eV, which are well-known as organic carbon C=C (1s → π*) and C-C (1s_ → _σ*) bonds^39^. The sharp peaks of Ca *L*2,3 edge at 351 eV and O-K edge at 548 eV were also detected, indicating the presence of calcium and oxygen at the interface. However, no carbonate group was observed, implying that the bond between the organic layer and calcite was not caused by carbonate bonding. A total of 15 EELS spectra were collected from different regions of the extracted zone of the sample (only three of them are shown). The observations confirm that organic layer is bound to calcite by the ionic bond between calcium (Ca^2+^) and oxygen (O^−^), which forms a constituent part of the carboxyl group (COO^−^) of oil acids or asphaltenes.

Elemental analysis of the organic layer was then captured in three points (Fig. 5 points 1, 2, 3) in order to study the homogeneity of the layer composition along its length. We observed that elemental composition of the organic layer remained the same throughout its length and the average concentration of elements was 96 ± 3 at.%, 2 ± 0.2 at.%, 1 ± 0.1 at.%, 1 ± 0.1 at.% for C, O, Si and S respectively. However, the registration of hydrogen by EDXS is impossible. Nevertheless, such large amount of carbon means that observed organic layers consisted most likely of hydrocarbons from oil, which were chemically adsorbed on the calcite by an ionic bond between Ca^2+^ and COO^−^.

## Discussion

Water condensation experiments were accomplished by controlling the chamber pressure and temperature. With the temperature being kept near 0 °C it was observed that water droplets started to form on carbonate surfaces at the pressure of 850 Pa after 1:32 minutes. In this kind of experiment, one of the possible errors of contact angle estimation can occur when water droplets merge with each other and form one large drop (Fig. 1). In this case, contact angles and surface wettability can be determined incorrectly, due to increase of the contact line between water and the surface. This problem can be resolved by performing the time-lapse experiments before measuring contact angles. To avoid further possible errors in the contact angle calculation caused by non-optimally oriented water droplets, we applied the procedure to extract the droplet from the image. Then, the geometrical dimensions of each droplet can be calculated more accurately (see contact angle evaluation section).

One principle limitation of condensation experiments in ESEM is that the experimental setup allows to investigate only the distilled water condensation from the microscope chamber. While such studies will not help wetting properties of brine or oil to be determined, it may be useful for the analysis of rock wetting tendency to pure water based fluids, for example when hydraulic fracturing fluids are tested^42,43^. In current work, this technology was first applied to identify the organic layers on carbonate surfaces. Indeed, it was observed that the surface covered by hydrocarbon layers had hydrophobic wetting properties, while pure calcite exhibited hydrophilic. This result was also confirmed by EDXS analysis in the corresponding points of the surface. Additionally, the EDXS spectra were acquired from a number of points in order to obtain reliable quantitative data. One of the disadvantages of EDXS is the inability to detect hydrogen atoms. Thus, results obtained for elemental composition of the organic layer (Fig. 6) are relative and the presence of hydrogen should be considered in further studies.

Cryo-FIB approach with a combination of S/TEM experiments outlined in this work provides a significant contribution in characterization of the organic layers on carbonate surfaces. Herein, the presence of hydrocarbon thin layer was proved by using the combination of microscopic techniques. The number of authors^44,45^ suggested that fatty acids and asphaltenes from oil had strong adsorption tendency to calcite and proposed that they could alter initial water-wet wettability of minerals towards more oil-wet, which in turn would impact on oil or water displacement processes. However, by now, there was a lack of information about properties of the adsorbed layers, because typically wettability was measured by standard methods on macro-scale, which are limited and cannot account for the influence of the organic layers on micro-scale wetting properties. While average wettability of a core sample was not addressed in this work, the calculated thickness of hydrophobic organic layer (180 ± 12 nm) can be used in fluids displacement models in order to evaluate in the more accurate way the key parameters of fluids multi-phase flow through pore throats, such as capillary pressure curves. Moreover, the results obtained from methods developed herein can be used in understanding the causes of wettability alteration in carbonates. Herein, we suggest that asphaltenes react with calcium ions by the ionic bond between calcium (Ca^2+^) and oxygen (O^−^) from the carboxyl group (COO^−^). Then, on the surface of this organic layer other hydrocarbons from oil start to adsorb. As a result, wettability changes towards more oil-wet and water-injection ceases to be effective. Furthermore, we expect that other carbonate reservoirs with the high asphaltenes content in oil will have a similar cause of the surface wettability modification.

## Conclusions

Microscopic approaches (Cryo-FIB, S/TEM, EDX, and EELS) were applied for characterization of the organic layer adsorbed onto carbonate surfaces. ESEM method was implemented in order to analyze the micro-scale wettability of the carbonate sample and influence of the organic layer on wetting properties of the surface. In this work, the following conclusions can be stated:

1. Water condensation experiments (ESEM) can be used for direct microscale wettability investigation of rocks. The measured contact angles of non-extracted carbonate surface was 93 ± 1°, while the measured contact angles for the same surface after toluene extraction were 160 ± 3° and 73 ± 1°, with small variations between contact angle values of right and left sides. Two values of contact angles represented different types of wetting properties in the sample after toluene treatment.
2. Cryo-FIB method was successfully implemented in the cross section preparation, allowing further S/TEM study of the organic layer on carbonate surface to be conducted.
3. EDXS analysis was used to determine the elemental composition and the thickness of the organic layer. Obtained elemental composition showed amounts of C (96 ± 3 at.%), O (2 ± 0.2 at.%), Si (1 ± 0.1 at.%) and S (1 ± 0.1 at.%) with small variations between three measuring points. The measured thickness of the organic layer is 180 ± 12 nm.
4. The EELS spectra can be used for determination of chemical bond between organic layer and calcite by comparing the spectra obtained from different zones of sample. The results showed that organic layer is bonded with calcite by Ca–O bond.

## Experimental Section

### Core samples

Carbonate rocks were sampled from the oil saturated zone of an oil carbonate reservoir. All of the cores consisted of 99.8% of calcite with small impurities of magnetite and quartz. Samples were cut and prepared for microscopic measurements.

### Core preparation

Original core samples were broken into several clumps. Then, clumps were flooded with kerosene and toluene consistently to remove initial crude oil from the cores until effluents became transparent. Once the extraction was finished, clumps were dried to a constant weight and then placed in vials under exhaust hood for 48 hours to complete solvent evaporation. Experiments were conducted with original core samples and the cores after toluene extraction.

### SEM and ESEM tests

**Figure 7:**
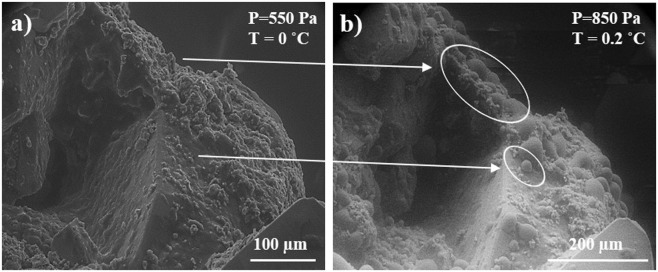
(**a**) ESEM image of initial carbonate rock sample. The sample temperature was 0 °C and chamber pressure was 550 Pa. (**b**) ESEM image of the same sample with formed water droplets. Micro-droplets of distilled water marked by the white circles. The sample temperature was 0.2 °C and the chamber pressure was 850 Pa.

Wetting properties of carbonate surfaces with hydrocarbon layers were analyzed using SEM/FIB Versa 3D DualBeam (FEI, USA), equipped with Schottky field emission gun and EDXS system (EDAX, USA). The study was conducted in high vacuum and ESEM modes. The images were obtained in the secondary electron (SE) mode using the gaseous secondary electron detector (GSED). ESEM experiment suggests that the sample is placed into the vacuum chamber of the SEM on a Peltier stage, which allows maintaining the sample temperature in a range between −25 °C and +25 °C. For water condensation on samples surface, the temperature was held near 0 °C, and the chamber pressure was varied in wide range between 600 Pa and 900 Pa in order to achieve dew point of water. It was experimentally observed that for sessile droplets formation pressure should be kept around 850 Pa and temperature of 0 °C. This is illustrated in Fig. 7, which provides the sequence of droplets growing on initial carbonate sample under conditions near the triple point of water: 0.1 °C, 850.1 Pa.

### Cryo-FIB

The experiments were carried out using Versa 3D FIB/SEM and Quorum PP3010T Cryo-FIB/SEM Preparation System (Quorum Technologies Ltd, UK) at a temperature below −140 °C. Samples were placed into vacuum chamber of the microscope on cryo-stage. Cooling of the latter was ensured by continuous circulation of liquid nitrogen. Cryo-FIB approach provides a unique opportunity to prevent structural damage during the preparation of thin cross sections of heat- sensitive materials. The cross section was prepared by Ga^+^ FIB and the sample was protected with 2 μm Pt layer deposited by e^−^ beam on the top of the area of interest before FIB procedure. Sequential thinning of the sample with decreasing of accelerating voltages from 30 kV to 2 kV of Ga^+^ was used to minimize amorphization.

### S/TEM

The cross-section of the sample was studied in a Titan 80–300 S/TEM (FEI, USA), equipped with a Schottky field emission gun, spherical aberration corrector (Cs probe corrector), energy dispersive X-ray spectroscopy (EDXS) system (EDAX, USA) and Gatan Image Filter (GIF) (Gatan, USA). The research was carried out at an accelerating voltage of 300 kV in bright field and dark field modes. A high angle annular dark field (HAADF) detector (Fischione, USA) was used for obtaining Z- contrast images in the STEM mode.

### EELS

The EELS spectra were obtained in STEM mode from thin layers of the specimen. Qualitative analyses were carried out by comparing the actual shape of the ionization energy loss for the measured points from sample areas, containing calcite and amorphous carbon^39–41^.

### Contact angle evaluation

**Figure 8:**
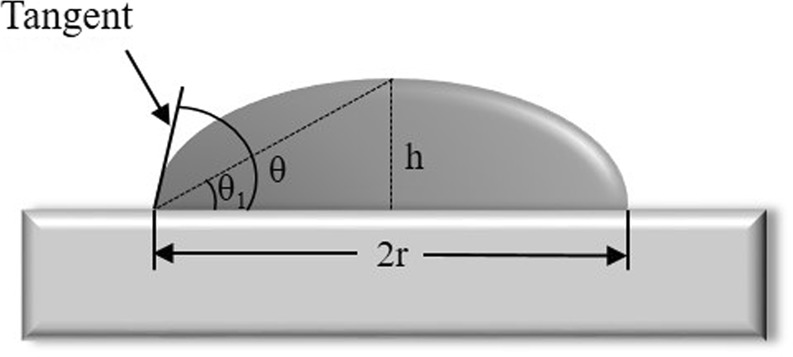
Drop shape analysis for contact angle evaluation.

Once the appropriate conditions for droplets formation were found, droplet dimensions were determined using Fiji platform^38^. Contact angles (θ) were calculated using the height (h) and the radius (r) of extracted droplet profiles (Fig. 8). Assuming that the droplet size is small, its profile can be approximated in the form of a circle. The following procedure was used to evaluate the contact angle on microscale (µm):

1. For the contact angles calculations the images of droplets sitting on the sample surface tilted respectively their normal were obtained.
2. After recording the images, the droplet profiles were cut from the SEM images using open-source image processing software ImageJ coupled with Fiji platform^38^.
3. After droplet profiles had been cut, they were rotated to horizontal position in the image coordinates (pixels), where the shape of the droplet was quite clear (Fig. 3c, for example).
4. Distance in pixels was then converted into the length dimension (µm) using ImageJ software.
5. Once optimal orientation and length conversion had been obtained, the height and radius of droplets were determined by measuring the length of corresponded edges of droplets using ImageJ software (Fig. 8, for example).
6. Once geometric parameters (h, r) had been detected, contact angles were calculated using the following equations:

1 $${\theta }_{1}={\tan }^{-1}\frac{h}{r},$$

2 $$\theta =2{\theta }_{1}$$

Wetting properties were defined by the contact angle value. The surface was considered to be hydrophobic if θ > 90° and hydrophilic if θ < 90°. Surfaces had mixed wetting properties if they consisted of both hydrophobic and hydrophilic zones.

## Data Availability

All data generated and analyzed during this study are included in this published article.
